# Supplementary material for: Effect of Polymer Substrate on Adhesion of Electroless Plating in Irradiation-Based Direct Immobilization of Pd Nanoparticles Catalyst
Source: Nanomaterials (Basel). 2022 Nov 21;12(22):4106. doi: 10.3390/nano12224106 (PMC9697518; doi:10.3390/nano12224106)
Supplement: Supplementary file 1 [file nanomaterials-12-04106-s001.zip › nanomaterials-2026327-supplementary.pdf]

Supplementary Material

Effect of polymer substrate on adhesion of electroless plating in irradiation-based direct immobilization of Pd catalyst

Naoto Uegaki *, Satoshi Seino *, Yuji Ohkubo and Takashi Nakagawa

Graduate School of Engineering, Osaka University, 2-1, Yamada-oka, Suita-shi, Osaka 565-0871, Japan
* Correspondence: uegaki-n18@mit.eng.osaka-u.ac.jp (N. U.); seino@mit.eng.osaka-u.ac.jp (S.S.); Tel.: +81-06-6879-7886

Contents

- Low-magnification SEM images of Pd/substrates ···························· Figure S1
- Exterior picture of Pd/PPS 10 vol% after electroless Cu plating without the acceleration step ··········· Figure S2
- SEM images of the ABS substrate surface before and after irradiation···························· Figure S3
- SEM images of AS substrate surface before and after irradiation···························· Figure S4

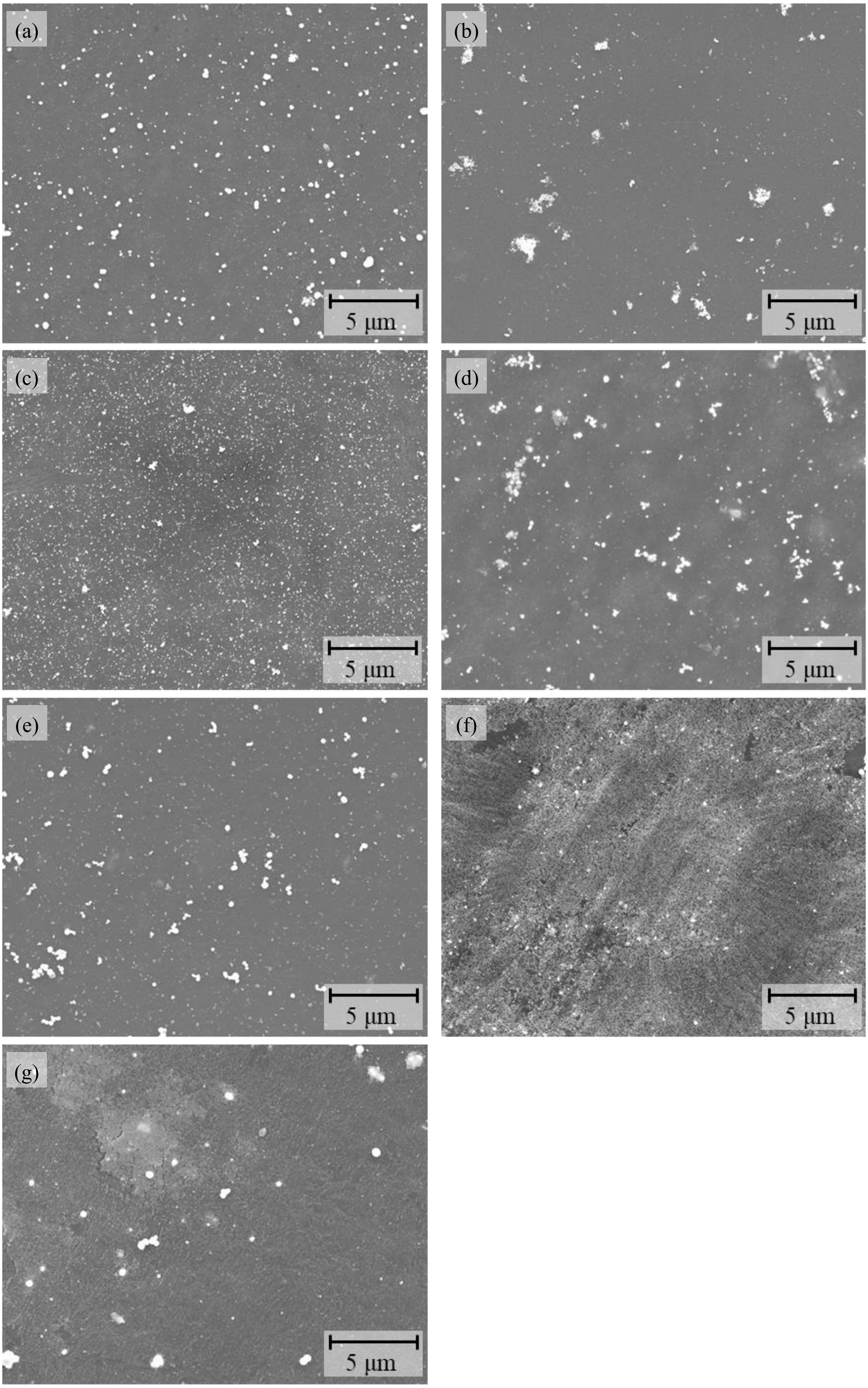

Figure S1. Low-magnification SEM images of Pd/substrates: (a) Pd/ABS, (b) Pd/AS, (c) Pd/PS, (d) Pd/PPS, Pd/PVC, Pd/PP, and (g) Pd/PE.

Pd nanoparticles were immobilized on PPS substrates using the method described in Section 2.1, with an aqueous solution of $Pd(NO_3)_2$ (1 mM) containing 10 vol% 2-propanol as the precursor solution. The obtained samples were denoted Pd/PPS 10vol%. Figure S2 shows exterior images of the samples after the electroless plating step without the acceleration step.

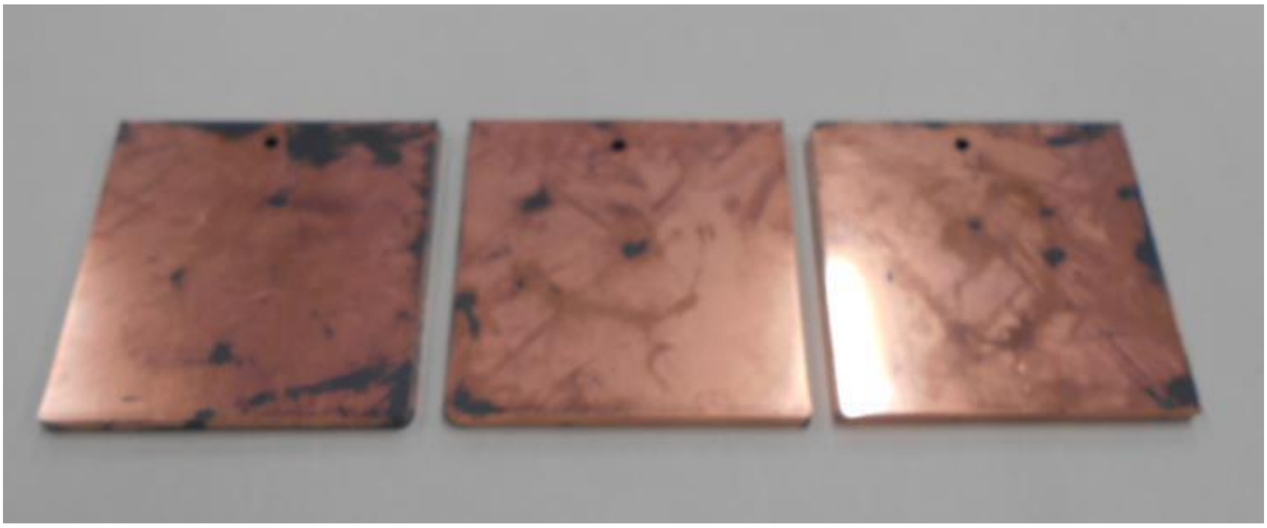

Figure S2. Exterior picture of Pd/PPS 10 vol% after electroless Cu plating without the acceleration step.

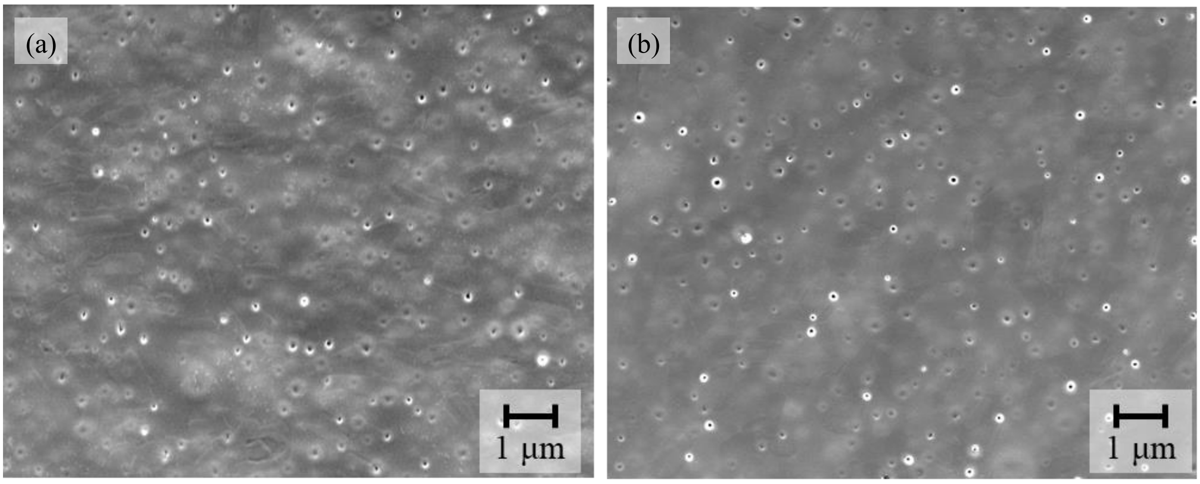

Figure S3. SEM images of the ABS substrate surface: (a) before irradiation and (b) after irradiation.

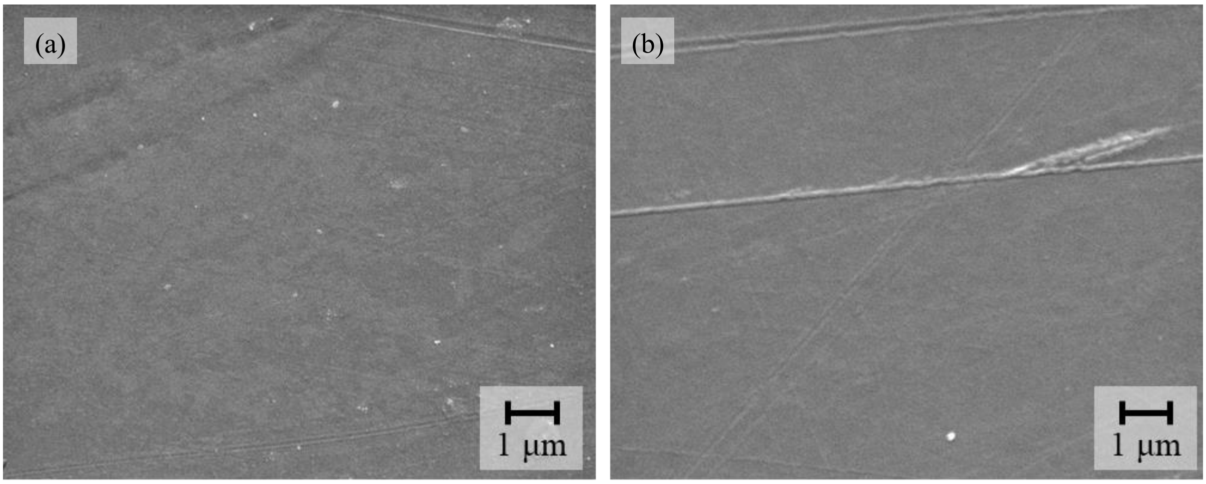

Figure S4. SEM images of AS substrate surface: (a) before irradiation, and (b) after irradiation.
